# Association between polyunsaturated fatty acid intake and infertility among American women aged 20–44 years

**DOI:** 10.3389/fpubh.2022.938343

**Published:** 2022-08-17

**Authors:** Ruohan Wang, Ying Feng, Jiahe Chen, Yingjiao Chen, Fang Ma

**Affiliations:** ^1^Center for Translational Medicine, Key Laboratory of Birth Defects and Related Diseases of Women and Children (Sichuan University), Ministry of Education, West China Second University Hospital, Sichuan University, Chengdu, China; ^2^Department of Obstetrics and Gynecology, West China Second University Hospital, Sichuan University, Chengdu, China; ^3^West China School of Basic Medical Sciences and Forensic Medicine, Sichuan University, Chengdu, China; ^4^Office for West China Institute of Women and Children's Health, West China Second University Hospital, Sichuan University, Chengdu, China

**Keywords:** infertility, docosapentaenoic acid, linoleic acid, polyunsaturated fatty acids, NHANES

## Abstract

**Background:**

Infertility is a nationwide public health priority in the U.S. However, few studies have investigated the effects of dietary intake of polyunsaturated fatty acids (PUFAs) on female infertility. This study explored the association between PUFA intake and risk of infertility.

**Methods:**

A total of 1,785 women aged 20–44 years from three National Health and Nutrition Examination Survey cycles (2013–2018) were included in this cross-sectional study. The intake of PUFAs was obtained from a 24-h dietary interview on two separate days with a 3–10-day interval, and nutrient residue models were used. Fertility status was assessed by positive response to two relative questions via a questionnaire. Logistic regression models were used and some covariates were adjusted.

**Results:**

Among all the participants, 340 (19.05%) women suffered from infertility. The intake of docosahexaenoic acid (DHA) (OR = 0.998, 95% CI 0.998, 0.009) was slightly related to the risk of infertility. In contrast, women with higher α-linolenic acid (ALA) (OR = 1.416, 95% CI 1.138, 1.763) and linoleic acid (LA) intake (OR = 1.020, 95% CI 1.002, 1.038) presented with a relatively higher risk of primary infertility. Furthermore, in 20–34-year-old women, higher omega-6/omega-3 was significant associated with the risk of infertility (OR = 1.002, 95%CI 1.000, 1.005).

**Conclusions:**

Our results suggest that PUFA intake is only slightly associated with infertility. The higher the DHA intake, the lower the risk of infertility regardless of age. In women with primary infertility, ALA and LA has negative effect.

## Introduction

Infertility is characterized by the failure to establish a clinical pregnancy after 12 months of regular, unprotected sexual intercourse or impairment of a person's capacity to reproduce, either as an individual or with his/her partner (1). According to the U.S. National Center for Health Statistics, it was estimated that ~6.7–19.4% of reproductive-aged women in the U.S. suffered from infertility between 2011 and 2019 (2, 3). The pathogenesis of infertility is complex, involving male and female factors, and a combination of both. In females, some common diseases such as polycystic ovary syndrome (4), endometriosis (5), and uterine fibroids (6) may cause infertility. To date, the U.S. Centers for Disease Control (CDC) declared the diagnosis and treatment of infertility as a national public health priority (7).

Diet is a modifiable lifestyle factor that plays an essential role in influencing human fertility (8). Various dietary components can have different effects on human physiological processes. Polyunsaturated fatty acids (PUFAs), a type of fatty acid containing multiple double bonds in their structures, mainly omega-3 and omega-6 fatty acids, are considered essential fatty acids because they cannot be produced by the human body. These fatty acids must be included in the daily diet. The most common sources of PUFAs are crop seeds, vegetable oils and cereal products, whereas for some types of PUFAs, such as eicosapentaenoic acid (EPA), docosapentaenoic acid (DPA), and docosahexaenoic acid (DHA), fish is the main source (9).

PUFAs play a significant role in the regulation of body homeostasis. Omega-3 fatty acids, especially EPA and DHA, exert anti-inflammatory, vasodilatory, bronchodilatory, and platelet anti-aggregation effects (10), whereas omega-6 fatty acids have opposing effects related to inflammation, vasoconstriction, and platelet aggregation (11). Persistent chronic inflammation leads to high risk of cancer and atherosclerosis, which can contribute to acute cardiovascular diseases (12, 13). In contrast, omega-3 fatty acids not only alter the functions of vascular and carcinogen biomarkers but also offer substantial protection against many chronic and metabolic diseases (14, 15). Thus, a proportionally higher intake of omega-3 fatty acids can protect against inflammatory diseases, cancer, cardiovascular diseases, diabetes, obesity, osteoporosis, neurological degeneration, and bone fractures (10, 13, 16, 17).

Furthermore, PUFAs are considered vital for reproductive health. In males, PUFAs can affect fertility (18). Previous researchers have found that DHA accumulated during sperm maturation in the sperm membrane (19) and was associated with higher sperm motility (20, 21), normal morphology (20, 22), and concentration (20, 23). Sperm membrane DHA can also affect key fertilization events such as capacitation, acrosome reaction, and sperm-oocyte fusion (24). In females, PUFAs are essential substrates that can affect female fertility during early reproductive phase, including oocyte maturation, embryo implantation, and oocyte quality (25–28).

There has been growing concern about the effect of dietary intake of fatty acids, especially omega-3 and omega-6 fatty acids, on the prevalence of female infertility. However, clinical research on dietary intake of PUFA and female infertility is still lacking. Associations between omega-3 and omega-6 fatty acids and body health have been explored mainly in *in-vitro* and animal studies, but the potential impact on female fertility needs attention. The objective of this study was to determine whether omega-3 and omega-6 fatty acids in the diet are associated with infertility using a national population-based survey of three National Health and Nutrition Examination Survey (NHANES) cycles (2013–2018) in representative American civilian women.

## Methods

### Data source and sample

A cross-sectional study was conducted based on data from 2013–2018 cycles of the NHANES, which aimed to assess the health and nutritional status of adults and children in the United States. Combining interviews and physical examinations, NHANES is a multistage study that uses complex stratified sampling methods to collect a representative sample of the U.S. population in every 2-year cycle. The NCHS Research Ethics Review Board (ERB) approved all the NHANES protocols.

**Figure 1:**
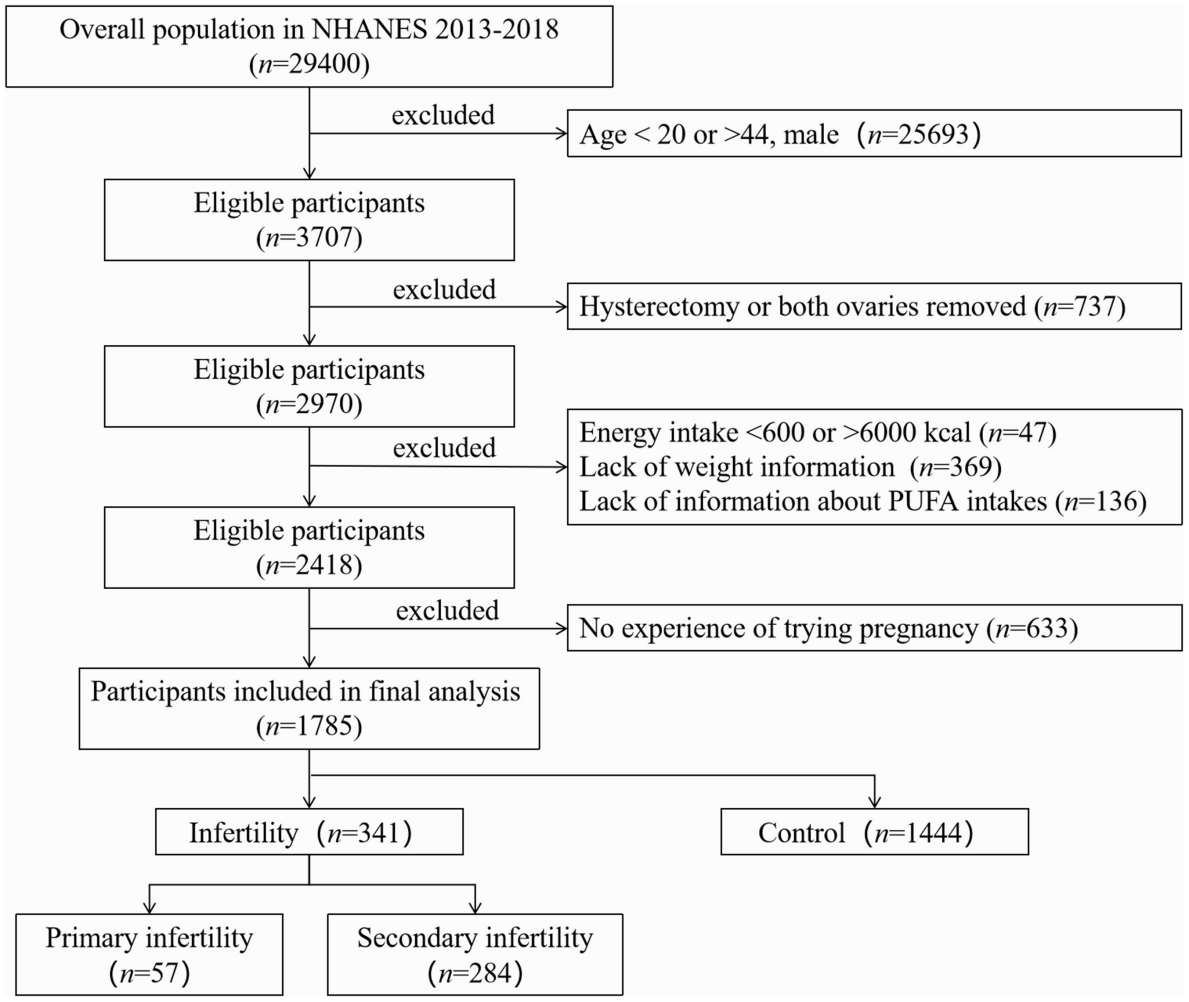
Flow chart of the sample selection from NEANES 2013–2018. According to the inclusion and exclusion criteria, 1,785 eligible participants were finally included in our study.

All female respondents between the ages of 20 and 44 years were eligible for this study (*n* = 3,707) because we can obtain available data on reproductive health from these age groups. Women with a history of hysterectomy or removal of both ovaries were excluded from the study. Women with kilocalorie intake per day <600 or >6,000 (29), or lack of information about PUFA intake and weight were excluded. Considering the possibility that some of these women may not have had the experience of trying pregnancy, we also excluded them from the study. A total of 1,785 women aged 20–44 years were included. The sample selection process is illustrated in Figure 1.

### Independent variables

The dietary data in NHANES was obtained from the dietary interview, which contained 24-h dietary recall interviews estimating the intake of energy, nutrients, and other food components from food and beverage consumption on two separate days with a 3–10-day interval. To minimize possible bias, all interviewers were required to complete a 1-week training and conduct supervised practice interviews before qualifying. In addition, a set of measuring guides (various glasses, bowls, and so on) was used, and additional foods not remembered earlier were asked repeatedly to ensure that the outcome was more reliable.

The Multiple Source Method (MSM) (https://msm.dife.de/) was used to estimate the usual dietary PUFA intakes of all participants from 24 h recall information. According to the User Guide of MSM, everyone was assumed as a habitual consumer. Age, race and BMI were included in MSM regression models as explanatory variables. There were five types of omega-3 fatty acids, including α-linolenic acid (ALA), stearidonic acid (SDA), EPA, DPA, and DHA, and two types of omega-6 fatty acids, including linoleic acid (LA) and arachidonic acid (AA). Nutrient residual models were used to estimate calorie-adjusted values for intake of each nutrient (30). In addition, we calculated the total omega-3 and omega-6 fatty acid intake, as well as omega-6/omega-3. Intakes of each PUFA were analyzed as a continuous variable as well as tertiles by dividing participants into three groups.

### Dependent variable

According to the definition of infertility (1), women whose response to either of two questions “Have you ever attempted to become pregnant over a period of at least a year without becoming pregnant?” “Have you ever been to a doctor or other medical provider because you have been unable to become pregnant?” was “yes” were considered ever infertile (31), which were further divided into primary infertility group and secondary infertility group based on the answer from another question “Ever been pregnant?”. Furthermore, if a person reported not ever infertile yet never had children, we considered that they might not have been trying pregnancy and excluded them (Figure 1).

### Covariates

Covariates were selected based on known associations between infertility and PUFA intake based on previous studies (31–33). Demographic variables such as age, race, educational level, marital status, ratio of family income to poverty (RIP), and smoking status were included in the analysis. In addition, reproductive factors such as age at the first menstrual period, regular periods, treatment for pelvic infection/pelvic inflammatory disease (PID), birth control pills, and female hormones were also included in the study.

### Statistical analysis

All statistical analyses were conducted according to the NHANES analytic guidelines (34). The appropriate dietary two-day sample weight was used to produce national and representative estimates, and all nutrients were adjusted by energy intake using nutrient residual models. Continuous variables with normally distributed were presented as mean and standard deviation (S. D.), otherwise median, P_25_ (25 percentile) and P_75_ (75 percentile). Categorical variables were presented as percentages. χ^2^ (for categorical variables), two independent sample *t*-tests (for normally distributed variables) and Wilcoxon rank sum test (for not normally distributed variables) were conducted to test the significance of the differences between the infertility and control groups.

The association between PUFA intake and infertility was assessed using two logistic regression models: a crude model (no covariate adjusted) and an adjusted model containing all covariates. Considering the effect of age on infertility, we conducted a subgroup analysis stratified by age (35 years). In addition, because of the possible differences between primary and secondary infertility, we further analyzed data from women with primary and secondary infertility separately. Odds ratio (OR) and 95% confidence intervals (CI) were used to assess the strength of the association. All statistical analyses were performed using SAS software version 9.4.

## Results

### Population characteristics of participants

A total of 1,785 women aged 20–44 years were included in this analysis, of which 340 (19.05%) suffered from infertility. The characteristics of all the participants are shown in Table 1. The results revealed that the infertile population in our study had higher education levels and family income, and they tended to be married. However, women with infertility showed higher BMI values. Among the reproductive factors related to infertility, pelvic infection and female hormones were more common in infertile population.

**Table 1:** Population characteristics of U.S. women aged 20–44 y, National Health and Nutrition Examination Survey, 2013–2018, weighted.

	**Overall**	**Infertility**	**Control**	***P* value**
	**(*n* = 1,785)**	**(*n* = 341)**	**(*n* = 1,444)**	
Age, y, mean (S.D.)	33.27 (6.86)	33.91 (6.96)	33.12 (6.84)	0.059
**Race**				
Mexican American	14.20	13.07	14.47	0.322
Other Hispanic	7.86	6.16	8.26	
Non-Hispanic White	51.34	55.78	50.30	
Non-Hispanic Black	16.14	13.70	16.72	
Non-Hispanic Asian	5.79	5.73	5.81	
Other Race - Including Multi-Racial	4.66	5.57	4.44	
**Education level**				
Less than 9th grade	4.31	2.77	4.67	0.001
9–11th grade	9.37	9.61	9.31	
High school graduate/GED or equivalent	22.53	15.82	24.11	
Some college or AA degree	36.83	37.23	36.74	
College graduate or above	26.96	34.56	25.18	
**Marital status**				
Married	54.23	59.89	52.90	<0.001
Widowed	0.81	2.78	0.35	
Divorced	7.37	6.72	7.53	
Separated	3.46	2.88	3.60	
Never married	19.16	16.42	19.80	
Living with partner	14.96	11.31	15.82	
RIP, mean (S.D.)	2.43 (1.62)	2.75 (1.65)	2.35 (1.60)	<0.001
**Smoking**				
Yes	34.01	36.86	33.34	0.219
No	65.99	63.14	66.66	
BMI, mean (S.D.)	29.10 (7.98)	30.98 (9.55)	28.65 (7.53)	<0.001
Menarche age, y, mean (S.D.)	12.55 (1.73)	12.44 (1.87)	12.57 (1.70)	0.252
Energy, kcal, mean (S.D.)	1887.44 (626.81)	1887.44 (626.81)	1902.90 (619.84)	0.613
**Regular periods**				
Yes	94.17	93.66	94.28	0.657
No	5.83	6.34	5.72	
**Pelvic infection**				
Yes	6.16	10.83	5.06	<0.001
No	93.84	89.17	94.94	
**Birth control pills taken**				
Yes	74.09	76.57	73.50	0.245
No	25.91	23.43	26.50	
**Female hormones taken**				
Yes	3.88	9.17	2.63	<0.001
No	96.12	90.83	97.37	

*RIP, the ratio of family income to poverty; BMI, body mass index*.

Furthermore, the comparisons between eligible participants (*n* = 3,707) and participants included in the final analysis (*n* = 1,785) regarding PUFA consumption, infertility and baseline information were presented in Supplementary Tables 1, 2. According to the results, age, education level, marital status, RIP, smoking status, BMI, regular period, acceptance of pelvic infection and infertility rate in eligible participants were significant different from women in the final analysis.

### Overview of omega-3 and omega-6 fatty acids intake

The average intakes of omega-3 and omega-6 fatty acids are shown in Table 2. Wilcoxon rank sum test showed that the average intake of DPA, LA, AA, total omega-6 fatty acid and omega-6/omega-3 in infertile women were significantly higher than those in the control group. In addition, the intakes of each PUFA were divided into three groups, and the ranges of tertiles were presented in Supplementary Table 3.

**Table 2:** Overview of omega-3 and omega-6 fatty acids intakes for U.S. women aged 20–44 years, weighted.

	**Overall (*n* = 1,785)**	**Infertility (*n* = 341)**	**Control (*n* = 1,444)**	***P* value**
ALA, g, M (P_25_, P_75_)	1.51 (0.67, 1.92)	1.46 (0.75, 1.93)	1.53 (0.67, 1.91)	0.781
SDA, mg, M (P_25_, P_75_)	6.32 (3.50, 13.98)	7.05 (3.64, 14.25)	6.21 (3.47, 13.92)	0.204
EPA, mg, M (P_25_, P_75_)	25.36 (14.12, 46.05)	27.17 (15.69, 49.07)	25.10 (13.99, 45.75)	0.078
DPA, mg, M (P_25_, P_75_)	27.66 (16.85, 48.49)	32.12 (19.30, 48.56)	27.11 (16.45, 48.43)	0.012*
DHA, mg, M (P_25_, P_75_)	58.12 (28.92, 125.32)	61.74 (27.99, 113.52)	57.32 (29.26, 130.92)	0.326
Total omega-3, g, M (P_25_, P_75_)	1.66 (0.89, 2.07)	1.61 (0.84, 2.06)	1.68 (0.91, 2.08)	0.899
LA, g, M (P_25_, P_75_)	17.56 (13.42, 27.10)	18.17 (13.97, 30.64)	17.37 (13.36, 26.41)	0.016*
AA, mg, M (P_25_, P_75_)	184.54 (114.14, 330.75)	206.75 (123.46, 368.20)	179.45 (111.25, 326.33)	0.047*
Total omega-6, g, M (P_25_, P_75_)	17.76 (13.58, 27.54)	18.31 (14.11, 31.17)	17.64 (13.48, 26.60)	0.017*
omega-6/omega-3, M (P_25_, P_75_)	9.51 (7.03, 14.81)	10.19 (7.45, 16.97)	9.45 (6.96, 14.29)	0.018*

^*^ P < 0.05. ALA, α-linolenic acid; SDA, stearidonic acid; EPA, eicosapentaenoic acid; DPA, docosapentaenoic acid; DHA, docosahexaenoic acid; LA, linoleic acid; AA, arachidonic acid.

### Associations between PUFA intakes and the risk of infertility

Table 3 demonstrates the association between PUFA intake and the risk of infertility in the participants. As for omega-3 fatty acids, in the crude model, those who consumed fewer DHA (OR = 0.999, 95% CI 0.998, 1.000) had a higher risk of infertility. When adjusted for all possible influential factors, the associations with DHA (OR = 0.998, 95% CI 0.998, 0.999) remained significant. When ALA was grouped into tertiles, compared with those in the lowest tertile, those in the middle tertile had a 33.9% (95% CI 0.434, 0.932) lower risk of infertility. In the population aged 20–34 years, when analyzed as continuous variables, only SDA presented protective effects of 1.4% (95% CI 0.973, 0.999). Furthermore, the upper tertile of DHA intake had a 38.9% (95%CI 0.386, 0.967) lower risk than the lowest tertile. In women over 35, DHA was still a slight protective effect (OR = 0.998, 95%CI 0.996, 0.999). For omega-6 fatty acids, in the overall population, LA (OR = 1.001, 95%CI 1.003, 1.018), AA (OR = 1.000, 95%CI 1.000, 1.001) and total omega-6 fatty acid (OR = 1.001, 95%CI 1.003, 1.018) presented the risk effects in the crude model, while in the adjusted model, only slight risk effects of AA (OR = 1.000, 95% CI 1.000, 1.001) were observed. Due to the strong collinearity between LA and total omega-6 fatty acids (data not shown), their effects remained the same. However, when stratified by age, no significant differences were found. In addition, we witnessed significant difference in the omega-6/omega-3 in 20–34-year-old women and controls, revealing a risk effect of 0.2% (95%CI 1.000, 1.005).

**Table 3:** Associations between PUFA intakes and the risk of infertility for U.S. women aged 20–34 years and 35–44 year, weighted.

	**Overall population (*****n*** = **1,785)**		**Women aged 20–34 years (*****n*** = **906)**		**Women aged 35–44 years (*****n*** = **879)**	
	**Crude model^a^**	**Adjusted model^b^**	**Crude model**	**Adjusted model**	**Crude model**	**Adjusted model**
**ALA**						
Continuous	1.027 (0.958, 1.102)	1.020 (0.950, 1.096)	0.987 (0.899, 1.084)	0.987 (0.898, 1.085)	1.064 (0.956, 1.183)	1.081 (0.968, 1.207)
Tertile 1	Reference	Reference	Reference	Reference	Reference	Reference
Tertile 2	0.661 (0.465, 0.941)*	0.661 (0.453, 0.996)*	0.626 (0.373, 1.050)	0.576 (0.327, 1.013)	0.674 (0.414, 1.096)	0.776 (0.462, 1.302)
Tertile 3	0.970 (0.746, 1.262)	0.925 (0.698, 1.226)	0.970 (0.657, 1.431)	1.024 (0.680, 1.542)	0.930 (0.648, 1.336)	0.859 (0.577, 1.278)
**SDA**						
Continuous	0.998 (0.993, 1.004)	0.995 (0.989, 1.002)	0.989 (0.978,1.001)	0.986 (0.973, 0.999)*	1.001 (0.995, 1.007)	0.999 (0.992, 1.006)
Tertile 1	Reference	Reference	Reference	Reference	Reference	Reference
Tertile 2	1.011 (0.714, 1.431)	0.976 (0.675, 1.411)	1.140 (0.720, 1.804)	1.207 (0.740, 1.969)	0.876 (0.513, 1.495)	0.746 (0.421, 1.323)
Tertile 3	1.164 (0.883, 1.534)	0.964 (0.715, 1.299)	0.774 (0.524, 1.145)	0.659 (0.430, 1.008)	1.696 (1.137, 2.529)*	1.374 (0.892, 2.116)
**EPA**						
Continuous	0.998 (0.996, 1.000)	0.998 (0.996, 1.000)	0.997 (0.994, 1.001)	0.997 (0.992, 1.002)	0.998 (0.996, 1.001)	0.998 (0.995, 1.001)
Tertile 1	Reference	Reference	Reference	Reference	Reference	Reference
Tertile 2	1.051 (0.715, 1.547)	1.021 (0.668, 1.559)	0.831 (0.498, 1.385)	0.781 (0.442, 1.379)	1.433 (0.788, 2.605)	1.317 (0.689, 2.519)
Tertile 3	1.201 (0.859, 1.679)	1.122 (0.775, 1.626)	0.795 (0.509, 1.243)	0.761 (0.462, 1.254)	1.908 (1.130, 3.221)*	1.665 (0.939, 2.952)
**DPA**						
Continuous	1.000 (0.997, 1.003)	0.999 (0.996, 1.002)	0.998 (0.992, 1.004)	0.997 (0.991, 1.003)	1.000 (0.997, 1.004)	0.999 (0.995, 1.003)
Tertile 1	Reference	Reference	Reference	Reference	Reference	Reference
Tertile 2	1.164 (0.764, 1.774)	1.017 (0.646, 1.601)	0.893 (0.503, 1.584)	0.832 (0.447, 1.549)	1.580 (0.824, 3.031)	1.307 (0.649, 2.630)
Tertile 3	1.458 (1.014, 2.098)*	1.264 (0.854, 1.871)	1.169 (0.732, 1.867)	1.075 (0.647, 1.785)	1.940 (1.075, 3.500)*	1.557 (0.827, 2.931)
**DHA**						
Continuous	0.999 (0.998, 1.000)*	0.998 (0.997, 0.999)*	0.998 (0.996, 1.000)	0.998 (0.995, 1.000)	0.999 (0.998, 1.000)*	0.998 (0.996, 0.999)*
Tertile 1	Reference	Reference	Reference	Reference	Reference	Reference
Tertile 2	0.898 (0.623, 1.294)	0.795 (0.535, 1.183)	0.742 (0.460, 1.198)	0.683 (0.406, 1.148)	1.177 (0.662, 2.091)	1.043 (0.553, 1.965)
Tertile 3	0.942 (0.688, 1.290)	0.772 (0.546, 1.091)	0.713 (0.469, 1.083)	0.611 (0.386, 0.967)*	1.282 (0.779, 2.109)	1.119 (0.643, 1.947)
**Total omega-3**						
Continuous	1.015 (0.947, 1.088)	1.068 (0.960, 1.189)	0.978 (0.892, 1.074)	0.997 (0.836, 1.142)	1.045 (0.941, 1.161)	1.107 (0.954, 1.285)
Tertile 1	Reference	Reference	Reference	Reference	Reference	Reference
Tertile 2	0.719 (0.505, 1.023)	0.884 (0.496, 1.578)	0.752 (0.459, 1.233)	0.526 (0.202, 1.375)	0.670 (0.405, 1.110)	1.199 (0.578, 2.488)
Tertile 3	0.961 (0.741, 1.246)	1.094 (0.724, 1.651)	0.901 (0.610, 1.331)	0.748 (0.396, 1.411)	0.955 (0.668, 1.365)	1.302 (0.744, 2.279)
**LA**						
Continuous	1.011 (1.003, 1.018)*	1.006 (0.997, 1.014)	1.015 (1.002, 1.028)	1.011 (0.997, 1.025)	1.008 (0.999, 1.017)	1.001 (0.990, 1.012)
Tertile 1	Reference	Reference	Reference	Reference	Reference	Reference
Tertile 2	1.006 (0.677, 1.494)	0.772 (0.500, 1.192)	0.958 (0.568, 1.618)	0.780 (0.438, 1.388)	1.096 (0.595, 2.020)	0.757 (0.389, 1.475)
Tertile 3	1.246 (0.902, 1.723)	0.939 (0.655, 1.346)	0.988 (0.645, 1.514)	0.782 (0.487, 1.257)	1.600 (0.960, 2.668)	1.044 (0.594, 1.835)
**AA**						
Continuous	1.000 (1.000, 1.001)*	1.000 (1.000, 1.001)*	1.000 (0.999, 1.001)	1.000 (0.999, 1.001)	1.000 (1.000, 1.001)*	1.000 (0.999, 1.001)
Tertile 1	Reference	Reference	Reference	Reference	Reference	Reference
Tertile 2	1.291 (0.859, 1.939)	1.140 (0.735, 1.767)	1.385 (0.796, 2.411)	1.377 (0.758, 2.503)	1.188 (0.652, 2.165)	1.003 (0.522, 1.929)
Tertile 3	1.412 (0.991, 2.013)	1.187 (0.812, 1.736)	1.273 (0.781, 2.074)	1.159 (0.683, 1.968)	1.538 (0.916, 2.584)	1.252 (0.718, 2.182)
**Total omega-6**						
Continuous	1.011 (1.003, 1.018)*	1.006 (0.997, 1.014)	1.015 (1.002, 1.027)*	1.011 (0.997, 1.025)	1.008 (0.999, 1.017)	1.001 (0.990, 1.012)
Tertile 1	Reference	Reference	Reference	Reference	Reference	Reference
Tertile 2	1.026 (0.691, 1.523)	0.776 (0.502, 1.197)	0.996 (0.590, 1.681)	0.785 (0.441, 1.397)	1.089 (0.592, 2.004)	0.757 (0.389, 1.472)
Tertile 3	1.248 (0.902, 1.727)	0.932 (0.650, 1.336)	1.003 (0.653, 1.539)	0.782 (0.487, 1.257)	1.572 (0.944, 2.617)	1.027 (0.585, 1.802)
**Omega-6/omega-3**						
Continuous	1.000 (0.999, 1.002)	1.000 (0.998, 1.002)	1.003 (1.001, 1.006)*	1.002 (1.000, 1.005)*	0.998 (0.993, 1.002)	0.999 (0.996, 1.002)
Tertile 1	Reference	Reference	Reference	Reference	Reference	Reference
Tertile 2	1.308 (0.916, 1.869)	1.282 (0.874, 1.881)	1.326 (0.811, 2.170)	1.336 (0.789, 2.262)	1.276 (0.759, 2.144)	1.345 (0.757, 2.390)
Tertile 3	1.321 (0.978, 1.784)	1.143 (0.829, 1.575)	1.298 (0.853, 1.974)	1.073 (0.687, 1.676)	1.308 (0.848, 2.018)	1.362 (0.843, 2.198)

OR (95% CI). ^*^ P < 0.05. ^a^Crude model: no covariate was adjusted. ^b^Adjusted model: age, race, educational level, marital status, the ratio of family income to poverty (RIP), smoking status, BMI, reproductive factors (age at first menstrual period, regular periods, treatment for a pelvic infection/pelvic inflammatory disease (PID), birth control pills, and female hormones taken). ALA, α-linolenic acid; SDA, stearidonic acid; EPA, eicosapentaenoic acid; DPA, docosapentaenoic acid; DHA, docosahexaenoic acid; LA, linoleic acid; AA, arachidonic acid.

### Associations between PUFA intakes and the risk of primary and secondary infertility

Considering the possible differences in factors between primary and secondary infertility, we further conducted a separate analysis in these two groups, and the results are displayed in Table 4. In this part, as for omega-3 fatty acid, ALA (OR = 1.416, 95% CI 1.138, 1.763) presented a relatively high-risk function in the primary population. Interestingly, when analyzed as tertiles, women with SDA intake in the second tertile had a 1.46-fold (95%CI 1.004, 6.063) higher risk of primary infertility than those in the first tertile. Similar to the results in the overall population, DHA was a slightly protective factor against the prevalence of secondary infertility (OR = 0.998, 95%CI 0.997, 0.999). However, when grouped as tertiles, significance can be found neither in primary infertility women nor in the secondary infertility population. The total omega-3 fatty acids only showed a positive effect in the primary infertility population in the crude model. With respect to omega-6 fatty acids, in the primary infertility population, relatively slight risk functions of LA (OR = 1.020, 95% CI 1.002, 1.038) and total omega-6 fatty acid (OR = 1.020, 95% CI 1.002, 1.038) intake were observed, whereas no significance was detected in women with secondary infertility. Furthermore, omega-6/omega-3 was not significant in either woman with primary or secondary infertility.

**Table 4:** Associations between PUFA intakes and the risk of primary and secondary infertility for U.S. women aged 20–44 years, weighted.

	**Primary population (*****n*** = **1,501)**		**Secondary population (*****n*** = **1,728)**	
	**Crude model^a^**	**Adjusted model^b^**	**Crude model^a^**	**Adjusted model^b^**
**ALA**				
Continuous	1.408 (1.170, 1.694)*	1.416 (1.138, 1.763)*	0.980 (0.911, 1.054)	0.963 (0.893, 1.038)
Tertile 1	Reference	Reference	Reference	Reference
Tertile 2	0.531 (0.222, 1.272)	0.464 (0.179, 1.205)	0.687 (0.472, 1.002)	0.662 (0.441, 0.995)*
Tertile 3	1.034 (0.582, 1,836)	0.829 (0.414, 1.658)	0.969 (0.730, 1.287)	0.856 (0.629, 1.165)
**SDA**				
Continuous	0.997 (0.984, 1.010)	0.994 (0.979, 1.009)	0.999 (0.993, 1.004)	0.995 (0.988, 1.002)
Tertile 1	Reference	Reference	Reference	Reference
Tertile 2	1.959 (0.863, 4.448)	2.467 (1.004, 6.063)*	0.884 (0.607, 1.286)	0.843 (0.566, 1.257)
Tertile 3	1.970 (0.969, 4.005)	2.044 (0.916, 4.564)	1.051 (0.785, 1.408)	0.874 (0.636, 1.200)
**EPA**				
Continuous	1.000 (0.997, 1.003)	1.001 (0.997, 1.004)	0.998 (0.995, 1.000)*	0.997 (0.994, 1.000)
Tertile 1	Reference	Reference	Reference	Reference
Tertile 2	1.674 (0.712, 3.938)	2.171 (0.821, 5.742)	0.943 (0.619, 1.436)	0.917 (0.578, 1.454)
Tertile 3	1.193 (0.535, 2.662)	1.448 (0.559, 3.752)	1.210 (0.845, 1.732)	1.139 (0.767, 1.691)
**DPA**				
Continuous	1.001 (0.995, 1.007)	1.003 (0.995, 1.011)	1.000 (0.996, 1.003)	0.998 (0.994, 1.002)
Tertile 1	Reference	Reference	Reference	Reference
Tertile 2	0.914 (0.354, 2.359)	0.711 (0.249, 2.028)	1.245 (0.789, 1.965)	1.073 (0.655, 1.756)
Tertile 3	1.319 (0.601, 2.897)	1.250 (0.522, 2.995)	1.491 (1.002, 2.217)*	1.277 (0.830, 1.963)
**DHA**				
Continuous	0.998 (0.996, 1.001)	0.998 (0.996, 1.001)	0.999 (0.998, 1.000)*	0.998 (0.997, 0.999)*
Tertile 1	Reference	Reference	Reference	Reference
Tertile 2	0.948 (0.429, 2.092)	0.945 (0.395, 2.258)	0.888 (0.597, 1.319)	0.815 (0.529, 1.257)
Tertile 3	0.859 (0.428, 1.721)	0.713 (0.323, 1.573)	0.966 (0.688, 1.356)	0.822 (0.565, 1.196)
**Total omega-3**				
Continuous	1.360 (1.142, 1.619)*	1.086 (0.674, 1.751)	0.968 (0.901, 1.041)	1.041 (0.932, 1.162)
Tertile 1	Reference	Reference	Reference	Reference
Tertile 2	0.612 (0.265, 1.414)	0.772 (0.138, 4.312)	0.741 (0.508, 1.082)	0.967 (0.527, 1.774)
Tertile 3	0.935 (0.526, 1.663)	0.492 (0.096, 2.525)	0.977 (0.739, 1.293)	1.149 (0.746, 1.770)
LA				
Continuous	1.021 (1.007, 1.036)*	1.020 (1.002, 1.038)*	1.009 (1.000, 1.017)*	1.001 (0.991, 1.011)
Tertile 1	Reference	Reference	Reference	Reference
Tertile 2	0.996 (0.452, 2.196)	0.583 (0.232, 1.466)	1.008 (0.650, 1.563)	0.759 (0.470, 1.227)
Tertile 3	0.813 (0.413, 1.598)	0.514 (0.228, 1.157)	1.375 (0.963, 1.964)	1.014 (0.682, 1.508)
**AA**				
Continuous	1.000 (0.999, 1.001)	1.000 (0.999, 1.002)	1.000 (1.000, 1.001)*	1.000 (0.999, 1.000)
Tertile 1	Reference	Reference	Reference	Reference
Tertile 2	1.533 (0.626, 3.750)	1.404 (0.538, 3.666)	1.242 (0.798, 1.933)	1.087 (0.674, 1.751)
Tertile 3	1.318 (0.587, 2.960)	0.878 (0.361, 2.136)	1.440 (0.982, 2.112)	1.237 (0.820, 1.866)
**Total omega-6**				
Continuous	1.021 (1.007, 1.035)*	1.020 (1.002, 1.038)*	1.008 (1.000, 1.017)*	1.001 (0.991, 1.010)
Tertile 1	Reference	Reference	Reference	Reference
Tertile 2	0.999 (0.453, 2.201)	0.585 (0.233, 1.473)	1.034 (0.667, 1.602)	0.762 (0.472, 1.231)
Tertile 3	0.809 (0.412, 1.591)	0.514 (0.228, 1.156)	1.380 (0.965, 1.973)	1.005 (0.676, 1.493)
**Omega-6/omega-3**				
Continuous	0.995 (0.989, 1.001)	0.998 (0.991, 1.005)	1.001 (0.999, 1.002)	1.000 (0.999, 1.002)
Tertile 1	Reference	Reference	Reference	Reference
Tertile 2	1.845 (0.703, 4.844)	1.765 (0.610, 5.102)	1.249 (0.857, 1.820)	1.285 (0.844, 1.956)
Tertile 3	2.731 (1.207, 6.176)*	2.562 (1.064, 6.170)*	1.175 (0.855, 1.615)	1.003 (0.711, 1.416)

OR (95% CI). ^*^ P < 0.05. ^a^Crude model: no covariate was adjusted. ^b^Adjusted model: age, race, educational level, marital status, the ratio of family income to poverty (RIP), smoking status, BMI, reproductive factors (age at first menstrual period, regular periods, treatment for a pelvic infection/pelvic inflammatory disease (PID), birth control pills, and female hormones taken). ALA, α-linolenic acid; SDA, stearidonic acid; EPA, eicosapentaenoic acid; DPA, docosapentaenoic acid; DHA, docosahexaenoic acid; LA, linoleic acid; AA, arachidonic acid.

### The main resources of PUFA

Further, according to the food codes provided by NHANES (https://wwwn.cdc.gov/Nchs/Nhanes/2017-2018/DRXFCD_J.htm), we summarized and listed the top five foods that contributed to PUFA intakes in Table 5. As for omega-3 fatty acids, the main resource was fish, while for omega-6 fatty acids, crop seeds, vegetable oils and some meat products were major sources.

**Table 5:** The main resources of PUFA.

	**Main resources**
ALA	Flax seeds
	Chia seeds
	Walnuts, excluding honey roasted
	Pistachio nuts, roasted, without salt
	Walnuts, honey roasted
SDA	Mackerel, cooked
	Herring, baked or broiled, added/no added fat
	Sardines, canned in oil
	Mackerel, canned
	Oysters, steamed
EPA	Mackerel, cooked
	Herring, baked or broiled, added/no added fat
	Anchovy, cooked
	Shrimp, dried
	Sardines, canned in oil
DPA	Pompano, baked or broiled, fat added
	Mackerel, cooked
	Swordfish, steamed or poached
	Porgy, baked or broiled, fat added
	Shrimp, dried
DHA	Mackerel, cooked
	Anchovy, cooked
	Herring, baked or broiled, added/no added fat
	Swordfish, steamed or poached
	Mackerel, canned
LA	Mayonnaise, regular
	Walnuts, excluding honey roasted
	Pistachio nuts, roasted, without salt
	Vegetable oil
	Sunflower seeds, plain, unsalted
AA	Egg, yolk only, cooked, fat added
	Beef liver, fried
	Pompano, baked or broiled, fat added
	Turkey or chicken sausage
	Egg, whole, fried no added fat

## Discussion

In this national cross-sectional study of associations between PUFA intake and infertility, we found that women with lower DHA intake had a higher risk of infertility, and ALA and LA was a risk factor for primary infertility.

As for the effects of total PUFA intakes to infertility in the overall population of infertile women aged 20–44 years, previous studies have reported inconsistent results regarding these relationships. A nested prospective case-control study conducted by Stanhiser et al. (35) suggested that there was no association between serum omega-3 or omega-6 fatty acids and infertility, even though they only tested the serum levels and not the dietary intake of fatty acids. Another study, similar to our results, showed that higher levels of serum omega-3 fatty acids, not omega-6 fatty acids, were significantly associated with a higher probability of clinical pregnancy in women undergoing assisted reproductive technology (ART) cycles (36). The average ages in these two studies were 33.3 and 34.8 years, respectively. Considering the various bioavailabilities of PUFAs, the intake of omega-3 fatty acids cannot perfectly represent their serum levels (9). This may be one of the reasons why researchers have obtained inconsistent results. When evaluating the intake of PUFAs rather than circulating levels, the latest prospective cohort study revealed that women aged 30–44 years taking omega-3 supplements had a 1.51 (95% CI 1.12–2.04) time higher probability of conceiving compared to women who did not (37). Another prospective cohort study in 2018 suggested that low omega-3 fatty acid intake was associated with reduced fertility, using time to pregnancy (TTP) as the outcome index (38). Nevertheless, the studies mentioned above only tested the total levels of omega-3 and omega-6 fatty acids. When evaluated separately, due to the complexity of the pathogenesis of infertility, our results only revealed a very slight significant association between DHA and infertility: the lower the DHA intake, the higher the risk of infertility, regardless of age. In women aged >35 years, the effects were weakened, which may be mainly caused by the decline of ovarian function or any other possible decline in the reproductive system. Women aged >35 years always face the challenge of declining ovarian reserve, oocyte number, and quality (39), all of which are the main causes of infertility, and the effects of diet may be weakened. To date, no clinical trial has evaluated the relationship between DHA and infertility, and only a few basic studies have revealed their potential effects. Hohos et al. (40) found a strong positive correlation between EPA, DPA, and DHA and primordial follicle numbers in mice, suggesting potential associations with ovarian reserve. Another study revealed that DHA can stimulate the proliferation and steroidogenesis of bovine granulosa cells (41), which may consequently promote reproduction. In women with polycystic ovary syndrome (PCOS), which is a vital factor of infertility, previous studies found that EPA and DHA may improve PCOS symptoms in rats by decreasing lipids and reducing weight and metabolic anomalies (42). A case-control study of Chinese women (43) also indicated the protective effects of consuming long-chain omega-3 PUFA, especially EPA and DHA, on PCOS. Nevertheless, according to our results, EPA and DPA may not be as efficient as expected in preventing infertility.

As omega-6 fatty acids may compete for enzymes in the metabolism of fatty acids with omega-3 fatty acids, affecting the conversion of ALA to EPA and DPA, and eventually to DHA, it was recommended that omega-6/omega-3 consumption should be reduced to 4 or lower (35). We also tested the effect of the omega-6/omega-3 intake ratio on infertility, revealing the risk effects in women under 35. Two independent prospective cohort studies conducted on American women who underwent IVF cycles did not obtain consistent results. Chiu et al. (36) found that the serum omega-6/omega-3 was not associated with ART outcomes. In contrast, Jungheim et al. (44) suggested that the increased LA/ALA ratio was related to incremental implantation and pregnancy rates. Notably, both cohort studies focused only on the serum levels of PUFAs. Generally, omega-3 fatty acids are thought to be anti-inflammatory, whereas omega-6 fatty acids are pro-inflammatory (45). Many chronic diseases, such as cardiovascular diseases, cancer, and autoimmune diseases, are related to increased levels of inflammatory factors such as IL-1β, IL-6, tumor necrosis factor (TNF), and C-reactive protein (CPR). Moreover, these factors may be induced by higher omega-6 fatty acid intake and lower omega-3 fatty acid intake (46). Inflammation is associated with many gynecologic diseases, including endometriosis (47), adenomyosis (48), PCOS (49), and uterine fibroids (48), all of which lead to decreased fertility. However, since the results of clinical studies are inconsistent, further RCT or larger-sample prospective cohort studies may be needed to determine the exact effect of the omega-6/omega-3 on infertility or ART outcomes.

Secondary infertility, the most common form of female infertility worldwide several years ago (50), is thought to have a pathogenesis distinct from primary infertility. Reproductive tract infections, which may cause secondary fallopian tube obstruction, are the main cause of infertility after primiparity, especially in regions with high unsafe abortion rates and poor maternity care (50). A case-control study conducted in Rwanda on women with secondary infertility revealed that obstetric events (such as a history of no prenatal care during the last pregnancy, early age of the first pregnancy, unwanted pregnancies, and stillbirths), HIV, and other sexually transmitted infections could all contribute to secondary infertility (51). Unlike the complex pathogenesis of primary infertility, secondary infertility seems much simpler, and some cannot be reversed by dietary adjustments. Consequently, when only secondary infertility participants were included, we found only a slight positive effect of DHA. Indeed, the positive function of DHA is generally attributed to its impact on oocyte quality and steroidogenesis (36). Women with a birth history always have relatively more normal ovarian function, which can be easily reversed by dietary adjustment.

In contrast, when excluding participants with secondary infertility, we observed relatively strong effects of ALA, LA, and total omega-6 fatty acids. A previous study indicated potential negative correlations between serum ALA levels and implantation rates in women undergoing IVF cycles (52). In this study, researchers found a positive association between elevated serum ALA levels and the presence of endometriosis, which is a known factor in infertility (52). Another prospective cohort study observed negative correlations between metaphase II oocytes and ALA levels in follicular fluid (53). Nevertheless, owing to the inconsistent results of ALA in primary infertility when analyzed as continuous variables and tertiles, the results should be interpreted cautiously. Similar to ALA, as a main source of omega-6 fatty acids, LA had adverse effects on oocyte development. A basic study found a decline in LA concentration when the follicle size increased (54). Further studies revealed that the negative effect of LA on oocyte maturation may be caused by the inhibition of the development of metaphase II oocytes and cumulus cell expansion. Treatment of cumulus-oocyte complexes with LA decreased the cleaved embryo rate and blastocyst yield (55). Meanwhile, an elevated level of LA causes a higher omega-6/omega-3, which may also be a risk factor for infertility.

To identify the external validation, we compared the baseline information, PUFA intakes and infertility information between all eligible participants and the final participates, suggesting some differences. As the participants included in the final analysis were selected *via* the inclusive and exclusive criteria, the final results may be more applicable for the population satisfying these criteria. For example, women with kilocalorie intake per day <600 or >6,000, or with a history of hysterectomy or removal of both ovaries may not suitable.

The present study has some limitations. First, owing to the cross-sectional study design of the NHANES, it is difficult to provide evidence of a causal relationship between PUFA intake and the risk of infertility. Therefore, it is impossible for us to determine which event, the intake of PUFAs or infertility, comes first. Some participants may have consciously changed their dietary patterns after being diagnosed with infertility. In addition, the assessment of PUFA intakes may cause bias. Because of the non-prospective study design, dietary data could only be collected based on memory, leading to recall bias. However, efforts were made to minimize this kind of bias: to use a set of measuring guides (various glasses, bowls) or ask for additional foods not remembered earlier, repeatedly, to ensure that the outcome was more reliable. Also, we did not include dietary supplements in that we can solely get the available information about the total PUFA supplement intake, not separated fatty acid supplement intakes, and very few people in the final analysis had PUFA supplement consumption.

Despite these limitations, our study had several strengths. First, we had a large sample size with nationwide representative samples, which can increase the strength of the evidence. Furthermore, the usual dietary intakes of PUFAs were estimated by MSM, which was a new statistical method for calculating intakes combining dietary intake data with supporting data such as age and sex *via* regression models. In this way, the estimation of usual dietary intake became more accurate. Additionally, the importance and functions of PUFAs nowadays are always considered in males, not females, and clinical studies are regarding the same are absent. Existing studies on PUFAs and female infertility have only focused on serum levels of PUFAs, or solely carried out basic research on animal models. However, it is obvious that with regard to dietary advice for women in their daily lives, the intake matters.

Infertility is a multifactorial disease with various etiologies. Our study concluded that DHA was a protective factor against infertility, and LA and total omega-6 fatty acids were risk factors for primary infertility in women. For women aiming to improve fertility *via* a daily diet or dietary supplements, these results may provide evidence and instruction. Despite these limitations, the clinical implications and conclusions of this study should be interpreted carefully.

## Data availability statement

Publicly available datasets were analyzed in this study. This data can be found at: https://www.cdc.gov/nchs/nhanes/index.htm.

## Ethics statement

The studies involving human participants were reviewed and approved by the NCHS Research Ethics Review Board (ERB). The patients/participants provided their written informed consent to participate in this study.

## Author contributions

RW, YF, and FM contributed to conception and design of the study. RW and JC organized the database and wrote the first draft of the manuscript. RW performed the statistical analysis. RW, YF, JC, and YC wrote sections of the manuscript. All authors contributed to the article and approved the submitted version.

## Funding

This work was supported by the National Natural Science Foundation of China, China (31771662 and 31470797).

## Conflict of interest

The authors declare that the research was conducted in the absence of any commercial or financial relationships that could be construed as a potential conflict of interest.

## Publisher's note

All claims expressed in this article are solely those of the authors and do not necessarily represent those of their affiliated organizations, or those of the publisher, the editors and the reviewers. Any product that may be evaluated in this article, or claim that may be made by its manufacturer, is not guaranteed or endorsed by the publisher.
