# Supplementary material for: Association between polyunsaturated fatty acid intake and infertility among American women aged 20–44 years
Source: Front Public Health. 2022 Aug 17;10:938343. doi: 10.3389/fpubh.2022.938343 (PMC9428268; doi:10.3389/fpubh.2022.938343)
Supplement: Supplementary file 1 [file Data_Sheet_1.docx]

Supplementary Table 1 Population characteristics of the total eligible participants (*n*=3707) and participants included in the final analysis (*n*=1785), weighted.

|  | Eligible participants (*n*=3707) | Participants included in the final analysis (*n*=1785) | *P* value |
| --- | --- | --- | --- |
| Age, y, M (P_25_, P_75_) | 31.00 (25.00, 38.00) | 34.00 (27.00, 39.00) | <0.001 |
| Race |  |  | 0.070 |
| Mexican American | 13.01 | 14.20 |  |
| Other Hispanic | 7.53 | 7.86 |  |
| Non-Hispanic White | 54.41 | 51.34 |  |
| Non-Hispanic Black | 13.80 | 16.14 |  |
| Non-Hispanic Asian | 6.82 | 5.79 |  |
| Other Race - Including Multi-Racial | 4.42 | 4.66 |  |
| Education level |  |  | <0.001 |
| Less than 9th grade | 3.35 | 4.31 |  |
| 9-11th grade | 7.84 | 9.37 |  |
| High school graduate/GED or equivalent | 19.64 | 22.53 |  |
| Some college or AA degree | 35.79 | 36.83 |  |
| College graduate or above | 33.29 | 26.96 |  |
| Marital status |  |  | <0.001 |
| Married | 45.84 | 54.23 |  |
| Widowed | 0.60 | 0.67 |  |
| Divorced | 6.44 | 6.74 |  |
| Separated | 2.95 | 3.11 |  |
| Never married | 40.54 | 26.84 |  |
| Living with partner | 13.64 | 14.07 |  |
| RIP, M (P_25_, P_75_) | 2.31 (1.16, 4.08) | 2.06 (1.03, 3.84) | 0.004 |
| Smoking |  |  | 0.025 |
| yes | 30.52 | 34.01 |  |
| no | 69.44 | 65.99 |  |
| BMI, M (P_25_, P_75_) | 26.60 (22.31, 32.61) | 27.37 (22.96, 33.83) | 0.001 |
| Menarche age, M (P_25_, P_75_) | 12.00 (12.00, 13.00) | 12.00 (12.00, 13.00) | 0.579 |
| Energy, kcal, M (P_25_, P_75_) | 1796.50 (1393.00, 2215.00) | 1832.00 (1444.50, 2262.00) | 0.012 |
| Regular periods |  |  | <0.001 |
| yes | 89.75 | 94.17 |  |
| no | 10.24 | 5.83 |  |
| Pelvic infection |  |  | 0.005 |
| yes | 4.81 | 6.16 |  |
| no | 94.71 | 93.84 |  |
| Birth control pills taken |  |  | 0.333 |
| yes | 72.62 | 74.09 |  |
| no | 27.25 | 25.91 |  |
| Female hormones taken |  |  | 0.352 |
| yes | 4.38 | 3.88 |  |
| no | 95.48 | 96.12 |  |
| Infertility |  |  | <0.001 |
| yes | 13.73 | 19.04 |  |
| no | 86.27 | 80.96 |  |

RIP, the ratio of family income to poverty; BMI, body mass index

Supplementary Table 2 Overview of omega-3 and omega-6 fatty acids intakes for the total eligible participants (n=3707) and participants included in the final analysis (n=1785), weighted.

|  | Eligible participants (*n*=3707) | Participants included in the final analysis (*n*=1785) | *P* value |
| --- | --- | --- | --- |
| ALA, g, M (P_25_, P_75_) | 1.52 (0.73, 1.98) | 1.51 (0.67, 1.93) | <0.001* |
| SDA, mg, M (P_25_, P_75_) | 6.55 (3.51, 13.88) | 6.32 (3.50, 13.98) | <0.001** |
| EPA, mg, M (P_25_, P_75_) | 25.41 (14.09, 46.96) | 25.36 (14.12, 46.05) | <0.001* |
| DPA, mg, M (P_25_, P_75_) | 28.05 (16.59, 48.56) | 27.66 (16.85, 48.49) | <0.001* |
| DHA, mg, M (P_25_, P_75_) | 59.96 (29.86, 127.85) | 58.12 (28.92, 125.32) | <0.001* |
| Total omega-3, g, M (P_25_, P_75_) | 1.68 (0.92, 2.14) | 1.66 (0.89, 2.07) | <0.001* |
| LA, g, M (P_25_, P_75_) | 18.50 (13.73, 28.99) | 17.56 (13.43, 27.10) | <0.001* |
| AA, g, M (P_25_, P_75_) | 189.49 (113.21, 339.45) | 184.54 (114.14, 330.75) | <0.001* |
| Total omega-6, g, M (P_25_, P_75_) | 18.75 (13.88, 29.38) | 17.76 (13.58, 27.54) | <0.001* |
| omega-6/omega-3, M (P_25_, P_75_) | 9.75 (7.15, 14.83) | 9.51 (7.03, 14.81) | <0.001* |

* *P*<0.05

ALA, α-linolenic acid; SDA, stearidonic acid; EPA, eicosapentaenoic acid; DPA, docosapentaenoic acid; DHA, docosahexaenoic acid; LA, linoleic acid; AA, arachidonic acid

Supplementary Table 3 The ranges of tertiles of omega-3 and omega-6 fatty acids intakes.

|  | Tertile 1 | Tertile 2 | Tertile 3 |
| --- | --- | --- | --- |
| ALA | -7.92, 1.52 | 1.52, 1.80 | 1.80, 6.94 |
| SDA | -24.23, 3.98 | 3.98, 6.40 | 6.40, 193.36 |
| EPA | -41.27, 11.24 | 11.24, 20.89 | 20.89, 780.20 |
| DPA | -17.49, 13.37 | 13.37, 22.36 | 22.36, 425.60 |
| DHA | -41.46, 22.85 | 22.85, 46.64 | 46.64, 1130.93 |
| Total omega-3 | -7.76, 1.63 | 1.63, 1.88 | 1.88, 7.75 |
| LA | -20.99, 12.47 | 12.47, 15.64 | 15.64, 91.85 |
| AA | -129.44, 96.01 | 96.01, 149.17 | 149.17, 2589.73 |
| Total omega-6 | -21.12, 12.58 | 12.58, 15.79 | 15.79, 92.57 |
| omega-6/omega-3 | -391.63, 7.00 | 7.00, 9.01 | 9.01, 1250.23 |

* *P*<0.05

ALA, α-linolenic acid; SDA, stearidonic acid; EPA, eicosapentaenoic acid; DPA, docosapentaenoic acid; DHA, docosahexaenoic acid; LA, linoleic acid; AA, arachidonic acid
